# Spontaneous spinal intradural hemorrhage in dengue fever: a case report

**DOI:** 10.1186/s13256-022-03451-2

**Published:** 2022-05-30

**Authors:** Rajeev Mohan Kaushik, Ranjit Kumar, Madhurima Kaushik, Manju Saini, Reshma Kaushik

**Affiliations:** 1 Department of General Medicine, Himalayan Institute of Medical Sciences, Swami Rama Himalayan University, Swami Ram Nagar, P.O. Jolly Grant, Dehradun, 248016 Uttarakhand India; 2 Department of Neurosurgery, Himalayan Institute of Medical Sciences, Swami Rama Himalayan University, Dehradun, Uttarakhand India; 3 Department of Ophthalmology, Himalayan Institute of Medical Sciences, Swami Rama Himalayan University, Dehradun, Uttarakhand India; 4 Department of Radiodiagnosis, Himalayan Institute of Medical Sciences, Swami Rama Himalayan University, Dehradun, Uttarakhand India

**Keywords:** Dengue, Paraplegia, Spinal intradural hemorrhage, Thrombocytopenia

## Abstract

**Background:**

Spontaneous spinal cord hemorrhage is extremely rare in dengue fever. We report a case of spontaneous spinal intradural hemorrhage in dengue fever associated with severe thrombocytopenia.

**Case presentation:**

A 48-year-old Indian woman presented with fever and body aches followed by acute onset of paraplegia with bladder and bowel dysfunction and loss of sensations below the level of the umbilicus. She had severe thrombocytopenia and positive dengue serology. Magnetic resonance imaging of the spine showed compression of the spinal cord due to intradural hematoma at the D7–D8 vertebral level. The patient received symptomatic treatment for dengue fever and steroids. Emergency D7–D8 laminectomy with excision of the clot and dural repair was done after stabilizing the platelet count with multiple platelet transfusions. The constitutional symptoms responded well to the treatment. There was good improvement in sensory symptoms but negligible improvement in paraplegia with a change in muscle power from grade 0/5 to grade 1/5 in the postoperative period. The patient was discharged from the hospital in a stable condition, but paraplegia showed little improvement during follow-up of 1 year.

**Conclusions:**

Spontaneous spinal cord hemorrhage can present as acute paraplegia in dengue fever. Failure to recognize this complication can delay initiating appropriate treatment with permanent loss of neurologic function.

## Introduction

Nontraumatic intradural extramedullary spinal hematomas are very rare. They may lead to spinal cord compression or cauda equina syndromes as per their location [1]. Many etiologies have been associated with spontaneous spinal subdural hemorrhage [2] such as bleeding abnormalities, vascular malformations, drug-induced anticoagulation, lumbar puncture [3], epidural anesthesia, and spinal surgery [4, 5].

Dengue virus constitutes an important arbovirus in tropical and subtropical regions [6, 7] with 75% of the global dengue burden present in South East Asia and the Western Pacific region [8]. Spontaneous intracranial hemorrhage is a dreaded complication in dengue fever and can occur due to thrombocytopenia, while spontaneous spinal cord hemorrhage is extremely rare in dengue fever.

Delay in diagnosis due to the rarity of this complication can lead to serious consequences. We report a rare case of spontaneous intradural hematoma in dengue fever associated with severe thrombocytopenia.

## Case presentation

A 48-year-old Indian woman presented with complaints of fever and body aches for 3 days, and pain in the lower abdomen with inability to move both lower limbs for 1 day. The patient had urinary incontinence and constipation. There was no history of seizures, recent vaccinations, diarrhea, or respiratory infection. The patient had not suffered from any significant illness in the past. There was no relevant family history. At presentation, the patient was febrile (oral temperature 102 °F) and had mild conjunctival suffusion. Higher mental functions, speech, and cranial nerves were normal with grade 0/5 power in both lower limbs. Lower limbs were hypotonic with absent deep tendon jerks and mute plantars on both sides. Sensation to light touch was diminished, while pain, temperature, and posterior column sensations were lost below the umbilicus (T10 level). A provisional diagnosis of acute flaccid paraplegia with bladder and bowel involvement due to acute transverse myelitis was considered.

**Fig. 1:**
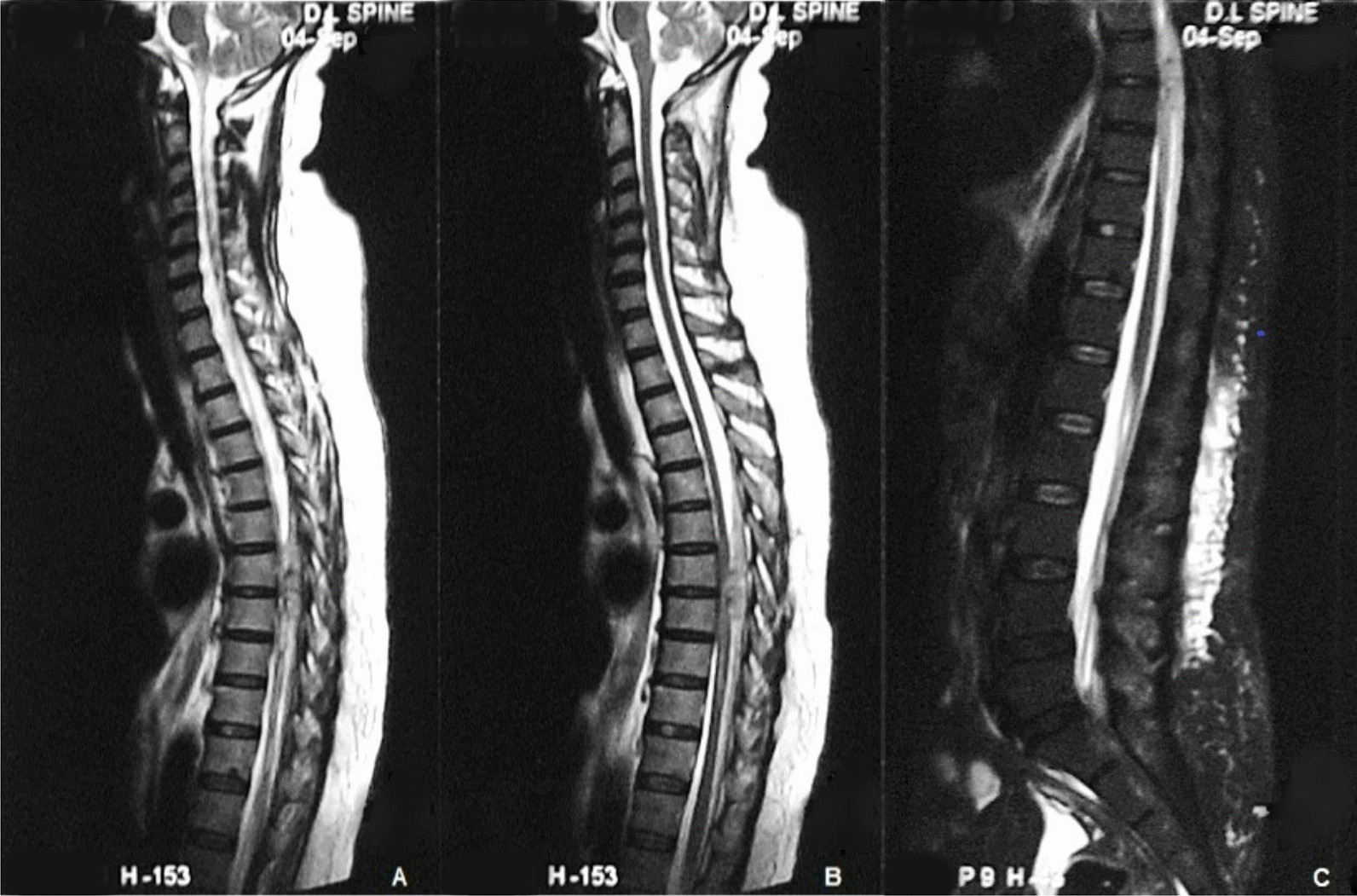
T2-weighted (**A** and **B**) and fat-suppressed T2-weighted (**C**) sagittal images showing intradural extramedullary hematoma at D7–D8 vertebral level

Laboratory investigations done outside the hospital had shown a rapid fall in platelet count from 130,000/mm^3^ to 12,000/mm^3^. Investigations in our hospital showed a normal white blood cell count and low platelet count (9410/mm^3^ and 40,000/mm^3^, respectively). Both peripheral blood smear and an immunochromatographic test (OptiMAL-IT, DiaMed AG, Cressier s/Morat, Switzerland) were negative for malaria along with a negative rapid test and IgM serology for scrub typhus. Dengue-specific NS1 antigen and IgG, and IgM antibodies were positive by a commercial micro-well enzyme immunoassay kit. Blood sugar, serum creatinine, serum electrolytes, and serum bilirubin were normal with raised aminotransferases. Bleeding time, clotting time, prothrombin time, international normalized ratio (INR), and activated partial thromboplastin time (aPTT) were normal. Ultrasonographic examination of the abdomen showed fatty liver and mild ascites with bilateral pleural effusion. Fundus examination was normal. Magnetic resonance imaging (MRI) of the dorsolumbar spine was suggestive of intradural, extramedullary hematoma at D7–D8 vertebral level (Fig. 1).

**Fig. 2:**
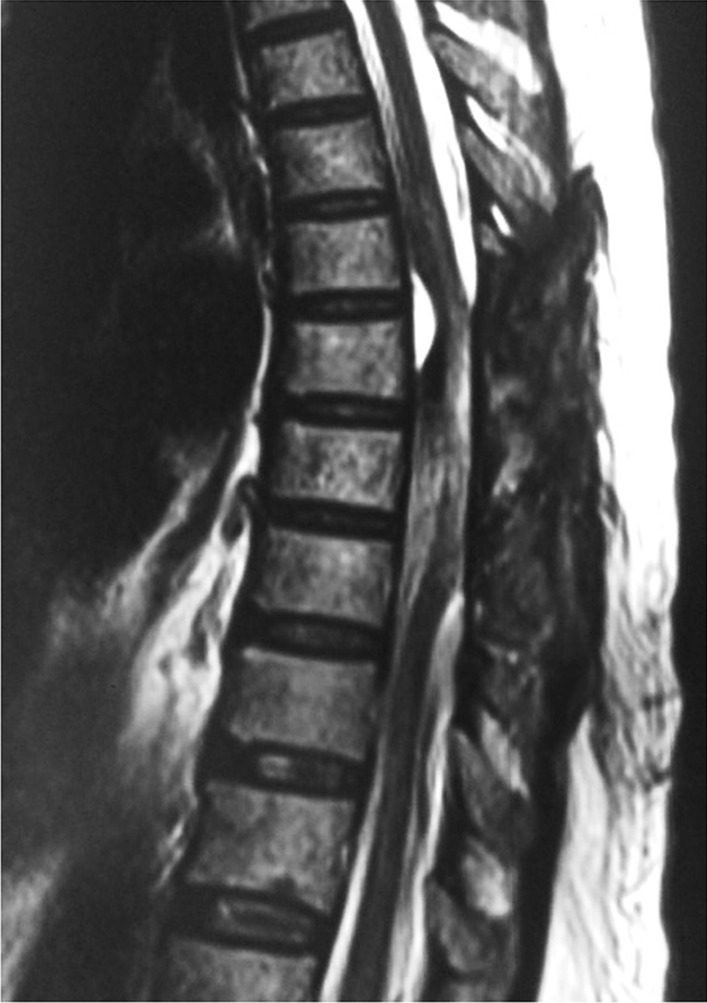
Postoperative T2-weighted sagittal image showing small hematoma in the anterior epidural space with cord edema at D7–D8 vertebral level

The diagnosis was revised to dengue hemorrhagic fever with thrombocytopenia with acute flaccid paraplegia due to compressive myelopathy caused by spontaneous spinal intradural hematoma at D7–D8 vertebral level. The patient received symptomatic treatment for dengue fever and intravenous methylprednisolone 1000 mg daily. She was given multiple transfusions of platelet concentrates and was taken for emergency surgery on the second day of hospitalization after raising the platelet count above the critical level. Intraoperative findings showed a cord bulge at D7–D8–D9 vertebral level with an intradural hematoma on the posterior and lateral aspects of the cord. Flimsy adhesions were present over nerve roots. D7–D8 laminectomy with excision of the clot and dural repair was performed. Repeat MRI examination of the dorsolumbar spine after 3 days was suggestive of a small hematoma in the anterior epidural space with cord edema with postoperative changes at the D7–D8 vertebral level (Fig. 2). Methylprednisolone was discontinued on the fifth day. The postoperative period was uneventful with good improvement in constitutional symptoms as well as sensory symptoms, but negligible improvement in paraplegia with a change in muscle power from grade 0/5 to grade 1/5. The patient was discharged in stable condition on the 15th day after surgery. At the time of discharge, the patient could accept oral feeds and her bladder and bowel functions had improved. She was advised limb strengthening exercises along with a foot splint. The patient showed marginal improvement in paraplegia during a follow-up period of 1 year.

## Discussion

Dengue hemorrhagic fever and dengue shock syndrome are the most severe sequelae of dengue infection. In 1976, neurological manifestations were initially reported as atypical manifestations of dengue infection [9]. Neurological manifestations have been reported in 0.5–20% of cases of dengue fever [10] and account for < 1% of the total systemic complications [11].

Neurological manifestations, such as headache, seizures, behavioral disturbances, and altered sensorium, can occur as part of encephalitis or aseptic meningitis in patients with dengue infection. Mononeuropathies, polyneuropathies, Guillain–Barre syndrome, myelitis, thrombosis, and intracranial hemorrhage have been reported previously in patients with dengue fever [11, 12]. Quadriparesis has been reported rarely due to myositis, Guillain–Barre syndrome [13, 14], and hypokalemia [15] in patients with dengue fever. Spontaneous spinal cord bleeding is extremely rare in dengue fever, and to the best of the authors’ knowledge, only three such cases have been reported in literature to date. Two cases had thrombocytopenia leading to extramedullary hematoma causing cervical cord compression with quadriparesis in one case [16] and spontaneous spinal subarachnoid hemorrhage causing paraparesis in the other [17], while the third one had normal platelet counts with quadriparesis due to hematomyelia that was attributed to platelet functional defect [18].

Our patient was a resident of a dengue-endemic area. Her clinical features and laboratory investigations were suggestive of the clinical picture of dengue fever, which was corroborated by positive serology for dengue virus infection. Acute onset of paraplegia with bladder and bowel involvement and a definite sensory level, in the presence of dengue virus infection, were in favor of noncompressive myelopathy in our patient. The possibility of spontaneous spinal cord hemorrhage causing compressive myelopathy was considered unlikely as it is extremely rare in dengue fever. However, the diagnosis of compressive myelopathy due to spontaneous intradural hematoma was established with the help of an MRI scan of the spine, which led to the neurosurgical intervention for spinal cord decompression. Early diagnosis and decompression of the spinal cord would have yielded a better neurologic outcome in our patient but could not be achieved due to delay in reporting to the hospital and the need to raise the platelet count to an optimum level before performing surgery.

## Conclusions

Paraplegia during dengue virus infection may not be solely due to Guillain–Barre syndrome, myositis, or hypokalemia, but may also be caused by compressive myelopathy due to spontaneous spinal cord hematoma, as seen in our case. This possibility should be considered by the treating physician, as misdiagnosis of dengue-related noncompressive myelopathy can delay initiation of appropriate treatment which may lead to an irreversible neurologic deficit.

## Data Availability

Not applicable.
